# Gross hematuria can be an impact of severe acute respiratory syndrome coronavirus 2 vaccination on immunoglobulin A nephropathy: a case report

**DOI:** 10.1186/s13256-022-03514-4

**Published:** 2022-07-11

**Authors:** Hiroshi Kanamori

**Affiliations:** Department of Nephrology, Fukuchiyama City Hospital, 231 Atsunaka-cho, Fukuchiyama, Kyoto 620-8505 Japan

**Keywords:** SARS-CoV-2 vaccine, COVID-19, Gross hematuria, Immune-mediated glomerulonephritis, IgA nephropathy

## Abstract

**Background:**

Immunoglobulin A nephropathy is typically accelerated by upper respiratory tract infections and can relapse following vaccination. There have been reports of patients who presented with immunoglobulin A nephropathy flares with or without gross hematuria following coronavirus disease 2019 vaccination. However, this relationship remains to be elucidated.

**Case presentation:**

Herein, we present the case of a patient with newly diagnosed immunoglobulin A nephropathy who presented with gross hematuria following the second dose of coronavirus disease 2019 vaccine. A 21-year-old Japanese woman presented with fever and new-onset gross hematuria 1 day after receiving the second dose of the coronavirus disease 2019 vaccine (Pfizer). She had microhematuria without proteinuria for 2 years at the time of her medical check-up. Gross hematuria resolved 6 days after the second dose of the coronavirus disease 2019 vaccine; however, microhematuria (> 100 per high-power field) and mild proteinuria were observed. She was admitted to our hospital 4 weeks after the second vaccination because of persistent urinary abnormalities. She was well before the vaccination and did not have any pulmonary involvement on chest radiography or any symptoms suggestive of coronavirus disease 2019. Renal biopsy revealed an immunoglobulin A nephropathy. The Oxford MEST-C score was M0E0S0T0C0. Our patient’s urinary abnormalities implied exacerbation of immunoglobulin A nephropathy after coronavirus disease 2019 vaccination.

**Conclusions:**

In our case, gross hematuria served as a trigger for immunoglobulin A nephropathy diagnosis, suggesting that nephrologists should pay close attention to gross hematuria and urinalysis after coronavirus disease 2019 vaccination.

## Background

It has been reported that immunoglobulin A nephropathy (IgAN) is typically accelerated by upper respiratory infections and can relapse after vaccination [1–3]. Most recently, there have been some reports of patients who presented with IgAN relapse, and a few reports of newly diagnosed IgAN following coronavirus disease 2019 (COVID-19) mRNA vaccination [4–7]. However, the relationship between IgAN and COVID-19 vaccination has not yet been fully elucidated. Herein, we report the case of a patient with newly diagnosed IgAN presenting with gross hematuria after receiving the second dose of Pfizer’s severe acute respiratory syndrome coronavirus 2 (SARS-CoV-2) mRNA vaccine.

## Case presentation

**Fig. 1:**
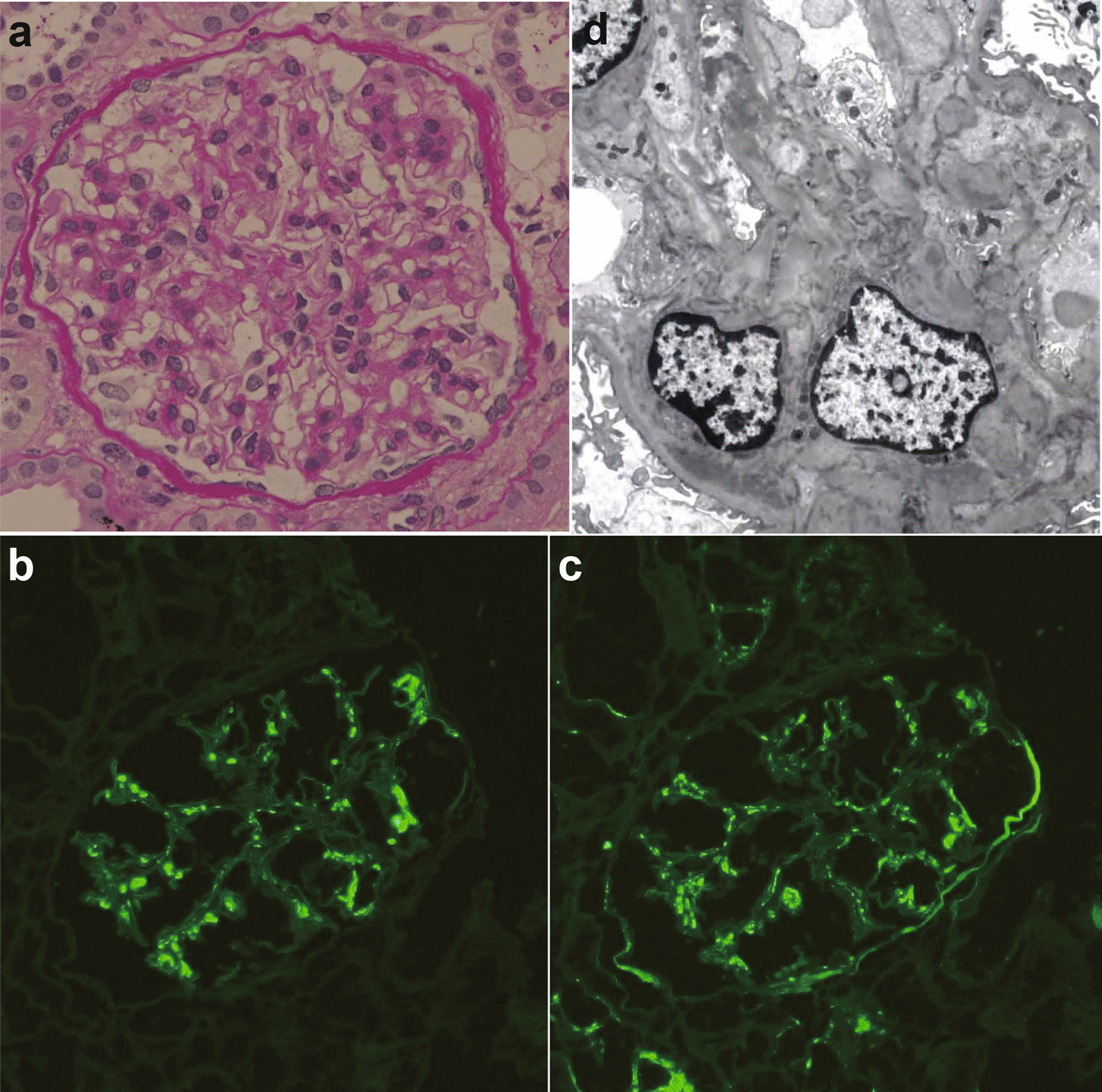
**a** Glomerular mesangial expansion and hypercellularity (periodic acid–Schiff, ×400). **b** Moderate-to-intense mesangial staining for IgA (immunofluorescence, ×200). **c** Moderate-to-intense mesangial staining for C3c (immunofluorescence, ×200). **d** Mesangial electron-dense deposits (electron microscopy, ×3000)

A 21-year-old Japanese woman presented with fever and new-onset gross hematuria 1 day after receiving the second dose of the COVID-19 Pfizer vaccine. She was well before the vaccination and did not have any pulmonary involvement on chest radiography or any symptoms suggestive of severe acute respiratory syndrome coronavirus-2 (SARS-CoV-2) infection throughout the COVID-19 pandemic. She was known to have not been infected with SARS-CoV-2, although neither serological nor polymerase chain reaction (PCR) testing was performed before and after vaccination. After receiving the first dose of the COVID-19 vaccine, she experienced muscle pain only at the site of injection. She had a medical history of microhematuria; the normal level of serum creatinine (0.60 mg/dL, reference 0.47–0.79 mg/dL) and microhematuria (3+, intense at qualitative score, reference negative) without proteinuria at the medical check-up 2 years ago. In her family history, her grandmother had hematuria (details unknown). Gross hematuria resolved 6 days after the second dose of the COVID-19 vaccine; however, microhematuria (> 100 per high-power field, reference 1–4 per high-power field) and mild proteinuria (3+, intense at qualitative score) with normal level of serum creatinine (0.64 mg/dL) were found. She was admitted to our hospital 4 weeks after the second vaccination because of persistent urinary abnormalities. Microhematuria (> 100 per high-power field) and mild proteinuria (urine protein-to-creatinine ratio, 0.15 g/g, reference < 0.15 g/g) were found, and the shape of red blood cells in urine was dysmorphic, which indicated glomerular hematuria. The serum creatinine level was normal (0.57 mg/dL). Physical examination results were normal, and her blood pressure was 123/74 mmHg. She was afebrile and had no lymphadenopathy, rash, throat erythema, or lower-extremity edema. At 4 weeks after the second vaccination, urinalysis showed microhematuria (> 100 per high-power field) and mild proteinuria (urine protein-to-creatinine ratio, 0.30 g/g). However, the normal range of proteinuria is 0.11 g/day (reference < 0.15 g/day). Laboratory data of serum showed albumin were within normal range (4.6 g/dL, reference 4.0–5.0 g/dL), and serum creatinine was also within normal range (0.53 mg/dL). Hemoglobin A1c (HbA1c) was within the normal range (5.4%, reference < 6.2%), and hepatitis B surface antigen (HBsAg) and hepatitis C antibody (HCV-Ab) tests were negative (HBsAg, negative; reference negative; HCV-Ab, negative; reference negative). Serum immunoglobulins were within normal limits (IgG: 1171 mg/dL, reference 880–1800 mg/dL, IgA: 246 mg/dL, reference 126–517 mg/dL, IgM: 95 mg/dL, reference 52–270 ng/dL), anti-streptolysin O antibody (ASO) and anti-nuclear antibody (ANA) were normal (ASO: 40 U/mL, reference < 250 U/mL, ANA: < ×40, reference < ×40), and complements were also within normal limits (C3: 93 mg/dL, reference 80–140 mg/dL, C4: 21.3 mg/dL, reference 11.0–34.0 mg/dL, CH50: 43 U/mL, reference 30–45 U/mL). Anti-neutrophil cytoplasmic myeloperoxidase antibody (MPO-ANCA), anti-neutrophil cytoplasmic proteinase 3 antibody (PR3-ANCA), and anti-glomerular basement membrane antibody (anti-GBM-Ab) were negative (MPO-ANCA: < 0.5 IU/mL, reference < 3.5 IU/mL, PR3-ANCA: < 0.5 U/mL, reference < 2.0 U/mL, anti-GBM-Ab: 0.6, reference < 7.0 U/mL). To confirm the diagnosis of glomerulonephritis, a renal biopsy was performed. Renal biopsy specimens (Fig. 1a–d) included 21 glomeruli, among which one had global sclerosis. In the remaining 20 glomeruli, one with increased cellularity of the mesangial cells and expansion of the mesangial area was observed (Fig. 1A; periodic acid–Schiff staining). Neither double contours nor spikes in the glomerular capillaries were observed. Segmental sclerosis, crescents, proliferation of endocapillary cells, and capsular adhesions were not observed. Tubular atrophy and interstitial fibrosis accounted for approximately 5% of the entire interstitium. Immunofluorescence microscopy (Fig. 1b, c) demonstrated diffuse moderate-to-intense deposits of IgA and C3c in mesangial lesions (Fig. 1b; IgA staining, Fig. 1c; C3c staining). IgG, IgM, and fibrinogen levels were extremely weak, and C4 and C1_q_ were negative. On the basis of these findings, the histological features were consistent with IgAN. Electron microscopy revealed electron-dense deposits in the mesangial and/or paramesangial areas (Fig. 1d). The Oxford MEST-C score (where M is mesangial hypercellularity, E is endocapillary hypercellularity, S is segmental sclerosis, T is tubular atrophy and interstitial fibrosis > 25%, and C is an active cellular or fibrocellular crescent) was classified as M0E0S0T0C0. At 6 weeks after the second vaccination, urinalysis showed no proteinuria (urine protein-to-creatinine ratio 0.09 g/g) and normal level of serum creatinine (0.56 mg/dL) was found; however, microhematuria (10–19 per high-power field) persisted.

## Discussion and conclusion

Urinary abnormalities of our patient implied exacerbation of IgAN after COVID-19 vaccination. Although the relationship has not been fully elucidated, COVID-19 vaccination may induce the production of anti-glycan antibodies against pathogenic IgA1 [4, 7]. In our case, gross hematuria triggered the diagnosis of IgAN.

IgAN is occasionally triggered and typically accelerated by upper respiratory infections [8]. SARS-CoV-2 can be associated with flares of underlying immune-mediated glomerulonephritis [9], and COVID-19 can trigger an IgA response in the bronchial mucosa [10]. Our patient was well and did not have any pulmonary involvement on chest radiography or any symptoms suggestive of SARS-CoV-2 infection. She was known to have not been infected with SARS-CoV-2. Therefore, we did not expect exacerbation of preexisting glomerulonephritis after the mucosal immune challenge in our case.

It has also been reported that IgAN can relapse after vaccination, including the influenza vaccine [1–3]. Our patient’s history of prior microhematuria, as noted in other reported cases where the COVID-19 vaccine was used [4–7], supports the idea that the immune response to vaccination, including the COVID-19 vaccine, activated a preexisting IgAN. The rapid development of gross hematuria immediately after the second dose of COVID-19 vaccine implicates a systemic cytokine-mediated flare, possibly via induction of a heightened IgA1 anti-glycan immune response. From this point of view, developing *de novo* antibodies may also lead to IgA-containing immune-complex deposits and the development of a new IgAN. However, in our case, preexisting microhematuria persisted, and it is natural to postulate that the prediagnostic IgAN was exacerbated by the second dose of COVID-19 vaccine.

On the other hand, it has been reported that IgAN flares following the second dose of COVID-19 vaccine without known prior exposure to SARS-CoV-2 is mediated by delayed-type hypersensitivity reactions via cell-mediated immune responses [5, 11].

In our case, it is unclear whether COVID-19 vaccination induced delayed-type hypersensitivity reactions and/or the production of anti-glycan antibodies for pathogenic IgA1, because neither the macrophage migration inhibition test nor investigation of serum IgA1 level was performed.

In terms of gross hematuria after COVID-19 vaccination in IgAN, there have been some recent reports to declare [4–7, 11–13]. All ten cases, including ours, are summarized in Table 1. The fact that gross hematuria developed soon after COVID-19 vaccination is not thought to be associated with age, sex, ethnicity, or the manufacturer of the vaccine (Pfizer or Moderna). Interestingly, except in one case, gross hematuria developed immediately after the second dose of the vaccine. This may imply that gross hematuria after the second dose of COVID-19 vaccine is mediated by delayed-type hypersensitivity reactions. To our knowledge, this is the first reported Japanese case of newly diagnosed IgAN in a kidney biopsy following the second dose of COVID-19 vaccination.

**Table 1:** Patient demographics and clinical characteristics

Patient	Age (years)	Sex	Race	MH	Medications	Vaccine	Episodes of gross hematuria before vaccination	Baseline (hematuria/uPCR/sCr)	Timing of gross hematuria after vaccination	Associated symptoms	Presentation after second dose (hematuria/uPCR/sCr)	Follow-up after second dose (hematuria/uPCR/sCr)	Reference
1	38	F	W	IgAN	RAASi	Moderna	Yes	(positive/0.63/well-preserved kidney function)	D1 after the second dose	Fever at D1	NA	(NA/1.40/well-preserved kidney function) 3 weeks after	4
2	38	F	W	IgAN	CY + Pred, then RAASi	Moderna	No	(positive/0.63/well-preserved kidney function)	D1 after second dose	Fever at D1	NA	(NA/0.40/well-preserved kidney function) 3 weeks after	4
3	52	F	A	IgAN	RAASi	Pfizer	Yes	(NA/0.6331/0.7–0.8)	D1 after second dose	Fever	(positive/2.4113/NA)	(resolved/1.441/NA) 5 days after	5
4	41	F	C	DM	NA	Pfizer	No	(NA/neg/NA)	D2 after second dose	Generalized myalgia at D2	(> 200/1.73/2.03)		6
5	30	M	WE/SA	None	None	Moderna	No	NA	D2 after second dose	Fever at D2	(3+/4+/1.02)	(positive/0.08/NA) 2 weeks after	7
6	NA	NA	NA	IgAN	NA	Pfizer	Yes	(NA/1.56/0.8)	D6 after second dose	Myalgia at D6	(positive/3.0/3.53)	(NA/baseline/baseline) 2 months after	11
7	NA	NA	NA	IgAN	NA	Pfizer	No	(NA/0.61/1.0)	D1 after second dose	Body aches	(positive/0.92/1.16)	(resolved/NA/NA) 5 days after	11
8	41	F	NA	IgAN	Tac, MPA, and steroids for KT	Pfizer	NA	(NA/0/NA)	D1 after first dose	Marked leukocytosis	NA	(NA/0.41/GFR 57 ml/min/1.73 m^2^)	12
9	50	M	NA	IgAN, HTN	None	Pfizer	NA	(11–25/2.40/1/17)	D2 after second dose	NA	(> 50/3.56/1.54)	(11–25/2.20/1.24)	13
Our case	21	F	Japanese	None	None	Pfizer	No	(3+/negative/0.60)	D2 after second dose	Fever at D2	(> 100/0.15/0.57)	(10–19/0.09/0.56) 6 weeks after	

*F* female, *W* white, *A* Asian, *C* Chinese, *M* Malay, *WE/SA* Western European and South American, *IgAN* IgA nephropathy, *DM* diabetes mellitus, *HL* hyperlipidemia, *HTN* hypertension, *MH* medical history, *RAASi* renin–angiotensin–aldosterone system inhibitor, *CY* cyclophosphamide, *Pred* prednisone, *Tac* tacrolimus, *MPA* mycophenolic acid, *KT* kidney transplantation, *sCr* serum creatinine (in mg/dL), *uPCR* urinary protein-to-creatinine ratio (in g/g), *NA* not applicable. Hematuria is expressed as the number of red blood cells per high-power field or qualitative score (1+, mild; 2+, moderate; 3+, severe) on urinalysis. Proteinuria is also expressed as a qualitative score (1+, mild; 2+, moderate; 3+, severe; and 4+, very severe) on urinalysis. None of the patients had episodes of gross hematuria before vaccination, and none was known to have been infected with severe acute respiratory syndrome coronavirus-2 (SARS-CoV-2), although neither serological nor polymerase chain reaction (PCR) testing was performed before and after vaccination

Although the issue that COVID-19 vaccination is closely associated with the new appearance of gross hematuria in IgAN remains to be elucidated, recent reports, including our case, emphasize the need for pharmacological adverse effects. When gross hematuria develops, nephrologists should first check for urinary tract infection and urological abnormalities. If these urological causes are ruled out, attention should be paid to glomerulonephritis, including IgAN. Vaccine providers and patients should be aware of this adverse effect. However, it should be emphasized that this is a relatively rare occurrence, and these reports should not lead to vaccine hesitancy during this pandemic because the benefits of vaccination would far exceed the exacerbation of preexisting glomerulonephritis.

In conclusion, COVID-19 vaccination can exacerbate IgA nephropathy; however the relationship between COVID-19 vaccination and exacerbation of IgA nephropathy is not fully understood. We suggest that nephrologists should pay close attention to urinalysis, including gross hematuria after COVID-19 vaccination.

## Data Availability

All data generated or analyzed during this study are included in this article.

## Abbreviations

IgAN: Immunoglobulin A nephropathy
COVID-19: Coronavirus disease 2019
SARS-CoV-2: Severe acute respiratory syndrome coronavirus 2
PCR: Polymerase chain reaction
HbA1c: Hemoglobin A1c
HBsAg: Hepatitis B surface antigen
HCV-Ab: Hepatitis C antibody
ASO: Anti-streptolysin O antibody
ANA: Anti-nuclear antibody
MPO-ANCA: Anti-neutrophil cytoplasmic myeloperoxidase antibody
PR3-ANCA: Anti-neutrophil cytoplasmic proteinase 3 antibody
anti-GBM-Ab: Anti-glomerular basement membrane antibody
